# A Brief Review of the Pharmacology of Amitriptyline and Clinical Outcomes in Treating Fibromyalgia

**DOI:** 10.3390/biomedicines5020024

**Published:** 2017-05-17

**Authors:** Kim Lawson

**Affiliations:** Department of Biosciences and Chemistry, Biomolecular Sciences Research Centre, Sheffield Hallam University, Sheffield S1 1WB, UK; k.lawson@shu.ac.uk; Tel.: +44-(0)114-225-3057

**Keywords:** fibromyalgia, amitriptyline, monoamine transporters, pain, fatigue, Fibromyalgia Impact Questionnaire

## Abstract

Fibromyalgia is a complex chronic condition characterized by pain, physical fatigue, sleep disorder and cognitive impairment. Evidence-based guidelines recommend antidepressants as treatments of fibromyalgia where tricyclics are often considered to have the greatest efficacy, with amitriptyline often being a first-line treatment. Amitriptyline evokes a preferential reduction in pain and fatigue of fibromyalgia, and in the Fibromyalgia Impact Questionnaire (FIQ) score, which is a quality of life assessment. The multimodal profile of the mechanisms of action of amitriptyline include monoamine reuptake inhibition, receptor modulation and ion channel modulation. Several of the actions of amitriptyline on multiple nociceptive and sensory processes at central and peripheral locations have the potential to act cumulatively to suppress the characteristic symptoms of fibromyalgia. Greater understanding of the role of these mechanisms of action of amitriptyline could provide further clues to the pathophysiology of fibromyalgia and to a preferable pharmacological profile for future drug development.

## 1. Introduction

Fibromyalgia is a complex chronic condition characterized by widespread pain, physical fatigue, non-restorative sleep and cognitive impairment [1,2]. A high incidence of co-morbidities, such as depression, irritable bowel syndrome, headache and temporomandibular joint dysfunction, with variable intensity are reported [1,2]. The worldwide prevalence of fibromyalgia, based on application of the American College of Rheumatology (ACR) 1990 criteria, has been reported to range from 0.4% to 8% of the population, with the condition being more common in females [3]. Fibromyalgia is therefore a major social and financial burden for patients and healthcare systems warranting effective and safe treatment options. Amplified responses of the central nervous system (CNS) to peripheral sensory input leading to enhanced neuronal excitability and central sensitization have been reported to be associated with hyperalgesia and allodynia, which are symptoms characteristic of fibromyalgia [1]. Thus, pharmacological treatments of fibromyalgia are usually focused on lowering pronociceptive excitatory neurotransmission and/or increasing antinociceptive neurotransmission in the CNS [2].

The evidence-based guidelines of the European League Against Rheumatism (EULAR) and the Association of Medical Scientific Societies in Germany (AWMF) recommend antidepressants, such as amitriptyline, duloxetine and milnacipran, as pharmacological therapies for fibromyalgia [4,5,6]. Although antidepressants are commonly used in the treatment of chronic pain, the efficacy varies depending on the type and mechanism of action of the drug and the specific condition being treated [7]. Antidepressants act on noradrenergic and serotonergic neurons, which are implicated in the mediation of endogenous pain inhibitory mechanisms and associated with several aspects of the pathophysiology of fibromyalgia [1,8]. Descending serotonergic-noradrenergic and opioidergic efferent pathways, which form the diffuse noxious inhibitory control (DNIC), are activated in healthy subjects by the application of intense painful stimuli leading to downregulation of the pain signal. In patients with fibromyalgia, the DNIC has been reported to be reduced or absent [9]. Consistent with decreased endogenous serotonergic and noradrenergic activity and a reduced DNIC in patients with fibromyalgia is an altered biochemistry of serotonin and noradrenaline. Lower levels of main metabolites of serotonin and noradrenaline, 5-hydroxyindoleacetic acid (5-HIAA) and 3-methoxy-4-hydroxyphenethylene glycol (MHPG) respectively, in cerebrospinal fluid and of l-tryptophan and serotonin in blood are observed in patients with fibromyalgia compared to healthy controls [1,8].

This review will focus on the pharmacology of amitriptyline that could be responsible for the benefits observed in the treatment of fibromyalgia.

## 2. Pharmacology of Antidepressants and Fibromyalgia

Serotonin reuptake transporters (SERT) and noradrenaline reuptake transporters (NET) are the main targets of antidepressants, such as tricyclic antidepressants (TCAs, e.g., amitriptyline), selective serotonin reuptake inhibitors (SSRIs, e.g., fluoxetine), serotonin and noradrenaline reuptake inhibitors (SNRIs, e.g., duloxetine and milnacipran) and selective noradrenaline reuptake inhibitors (NRIs, e.g., reboxetine). The affinities for monoamine reuptake transporters and selected neurotransmitter receptors of antidepressant drugs tested as treatments of fibromyalgia are summarized in Table 1 [10,11,12,13,14,15,16,17,18,19,20,21]. These antidepressants, through pharmacological actions on monoamine reuptake transporters, can facilitate the endogenous pain control system at neuroanatomical structures such as the locus coeruleus nucleus, the dorsal and magnus nuclei in the raphe, and the dorsal horn of the spinal cord [22]. A pathway from the midbrain periaqueductal gray (PAG) through the ventromedial medulla (VMM) to the dorsal horn constitutes a putative endogenous nociceptive modulatory system [23]. Evidence supports the PAG and VMM having multiple and coordinated effector functions that are not limited to nociceptive modulation but may involve homeostatic functions such as sleep-awake cycle, micturition and blood pressure [23]. A reciprocal feedback exists between the raphe nuclei and the suprachiasmatic nuclei, contributing to circadian rhythms and alteration of serotonin levels controlling sleep-awake states [24]. The locus coeruleus is a principal area for the synthesis of noradrenaline in the brain which, in addition to involvement in pain and stressor responses, mediates arousal of the sleep-awake cycle [25]. Consequently, many of the sympathetic effects during stress are mediated by the locus coeruleus increasing noradrenaline secretion and the raised noradrenaline levels alter, via a multimodal process, cognitive function through the prefrontal cortex [25,26]. Thereby, modulation of serotonin and noradrenaline levels, particularly within the descending processes, by the action of antidepressants on the monoamine reuptake transporters, has the potential of changing, in addition to pain, many of the characteristic symptoms of fibromyalgia. The involvement of serotonin and noradrenaline levels in the efficacy of antidepressants in fibromyalgia identifies 5-hydroxytryptamine (5-HT) and adrenergic receptors as playing a significant role in antinociceptive properties. Affinity for and/or efficacy at 5-HT and adrenergic receptors exhibited by antidepressants could influence the pain reducing profile but also result in unwanted effects [27,28]. Although action at muscarinic and histamine receptors of antidepressants has been related to the adverse cardiotoxicity and gastrointestinal disturbance, and sedative properties respectively, antinociceptive effects may also be achieved by actions at these receptors [27,28]. The efficacy of antidepressant drugs tested as treatments of fibromyalgia on the characteristic symptoms and quality of life assessments are summarized in Table 2 [29,30,31,32,33,34,35,36,37,38,39,40,41,42,43,44,45,46,47,48,49,50,51,52,53,54,55,56,57,58,59,60,61,62,63,64,65,66,67,68,69,70].

The optimum analgesic action of antidepressants in the treatment of fibromyalgia, as with other chronic pain conditions, is usually achieved with a dose lower than that required to achieve mood enhancement indicative of intrinsic analgesic efficacy [22]. Although the analgesic response is distinct from the effects on mood as indicated by the variation in analgesic efficacy among the chemical classes, many of the antidepressants over the dose range used in the treatment of fibromyalgia also reduce anxiety and depression scores (Table 2). Antidepressants exhibit diverse pharmacological properties with individual agents (within a class) exhibiting such effect to variable degrees, and this may account for possible differing outcomes in the treatment of fibromyalgia.

## 3. Pharmacology of Amitriptyline and Fibromyalgia

Of the range of antidepressant classes (TCAs, SNRIs, SSRIs, NRIs), TCAs are often considered to have the greatest analgesic efficacy, with amitriptyline (10–50 mg/day) being recommended as a first-line treatment for fibromyalgia [4,5,6]. The use and acceptance of TCAs in patients with fibromyalgia will be enhanced by the adverse effects often reported for this class of drugs occurring less commonly and with less severity when used as analgesics than as antidepressants [22].

**Figure 1:**
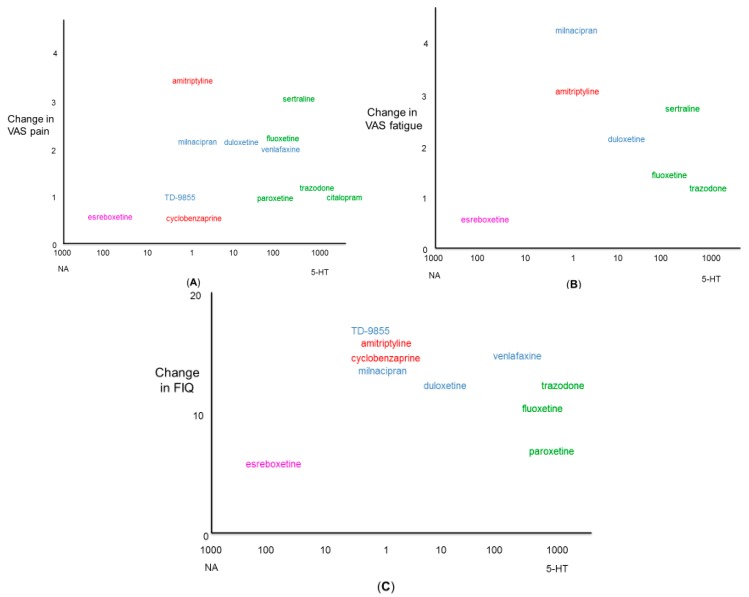
Schematic of monoamine transporter selectivity and efficacy of antidepressant drugs to reduce pain (**A**), fatigue (**B**) and Fibromyalgia Impact Questionnaire (FIQ) score (**C**) in patients with fibromyalgia. Selectivity ratios for the antidepressant drugs were determined from affinity and inhibitory properties for noradrenaline reuptake transporter (NET) and serotonin reuptake transporter (SERT) where NA indicates a selectivity for NET over SERT values and 5-HT indicates a selectivity for SERT over NET (see Table 1). Pain measurement (**A**) is presented as change on a 0–10 scale visual analogue scale (VAS); fatigue measurement (**B**) is presented as change on a 0–10 scale VAS; FIQ score (**C**) is presented as a change on a 0–100 scale (see Table 2). TCAs are indicated in red, SNRIs in blue, SSRIs in green and NRIs in purple.

A preferential outcome in the reduction of pain and fatigue associated with fibromyalgia and in the Fibromyalgia Impact Questionnaire (FIQ) score, which reflects a quality of life assessment, was observed with amitriptyline (10–100 mg/day) compared to the majority of other antidepressants (Figure 1; Table 2). The efficacy of amitriptyline relative to other classes of antidepressants (e.g., SNRIs, SSRIs and NRIs) infers that modulation of monoamine reuptake mechanisms and additional pharmacological properties are required to control the symptoms of fibromyalgia.

Although amitriptyline evokes a relatively balanced inhibition of serotonin and noradrenaline reuptake, the main metabolite, nortriptyline, preferentially inhibits noradrenaline reuptake (Table 1) [70]. Thus, in a clinical setting, the net outcome of amitriptyline treatment will place an emphasis on inhibiting noradrenaline reuptake, which may be relevant to changes in the biology of patients with fibromyalgia. For example, an increased release of substance P, a mediator of C fibres which contributes to central sensitization, is expressed as hyperalgesia if noradrenergic neuronal activity or the levels of noradrenaline decrease [71]. In patients with fibromyalgia, substance P levels are raised in the cerebrospinal fluid which could induce pain through the activation of neurokinin 1 receptors [8]. In addition to inhibiting presynaptic reuptake of noradrenaline and serotonin, amitriptyline has affinity for α-adrenergic, histamine, muscarinic cholinergic, 5-HT, *N*-methyl-d-aspartate (NMDA) and opioid receptors (Table 1) [12]. 

Although pain in patients with fibromyalgia is probably mediated by alternating activity levels in the CNS, associated with hyperexcitability, peripheral nociceptive generators enhancing the symptoms also play a role in the pathophysiology and need to be considered in the management of the condition [1,2,8]. A peripheral analgesic effect of antidepressants has been proposed [72]. Inhibition of the monoamine reuptake transporters, however, is unlikely to be the mechanism of action responsible for the analgesic properties of amitriptyline, because noradrenaline and serotonin enhance nociceptive transmission at a peripheral level. Thus, blockade of adrenoceptors and 5-HT receptors, and possibly of histamine and muscarinic receptors, as observed with amitriptyline but not SNRIs and SSRIs, is a more likely contributory analgesic mechanism of action at a peripheral level in patients with fibromyalgia [22]. Peripheral adenosine A1 receptors have also been proposed to be involved in systemically administered amitriptyline-induced antinociception [73]. Amitriptyline and other TCAs inhibit the neuronal uptake of adenosine which would lead to enhanced stimulation of adenosine receptors at both peripheral and CNS levels producing analgesia [28]. Interestingly, raised adenosine levels associated with d-ribose treatment have been reported to reduce symptoms in patients with fibromyalgia [74].

Although amitriptyline has been demonstrated to bind to opioid receptors and NMDA receptors, interaction with these receptors at therapeutic drug concentrations has been the subject of debate [70]. Antidepressants binding to the NMDA receptor reduce glutamate-induced intracellular calcium accumulations which would lead to suppression of cell excitation [75,76]. The neuronal hyperexcitability that is observed in fibromyalgia, causing both spontaneous and stimulus-evoked pain, may be a consequence of heightened sensitivity of the NMDA receptor-operated ion channels, which could have an impact on the concentrations of amitriptyline evoking a therapeutic effect [77]. The opioidergic system also appears to be involved in the mechanism of action of certain antidepressants that have antihyperalgesic action, such as TCAs, but not those with antinociceptive effects (e.g., duloxetine), which is an analgesic profile that would be relevant to the symptoms characteristic of fibromyalgia [78].

At therapeutic drug concentrations, amitriptyline also interacts with sodium, calcium and potassium channels. TCAs block open and inactivated states of sodium channels by binding to the local anaesthetic receptor, a property that may contribute to the analgesic efficacy in patients with fibromyalgia [79]. Although the action at potassium channels has been suggested to be involved in the adverse effects of amitriptyline (e.g., cardiac events), the role of interaction with specific calcium and potassium channel subtypes at therapeutic concentrations with the beneficial effects in humans needs further investigation. Central antinociception induced by amitriptyline has been reported to involve the opening of different subtypes of K^+^ channels (voltage-gated Kv1.1, K_ATP_ and Ca^2+^-gated) which represent important intracellular effectors in the analgesic activity of TCAs [80]. Further, amitriptyline has an affinity for the Kv7 family of potassium channels, which are the targets of potential treatments of fibromyalgia [81,82].

Dopamine deficiency in the CNS has also been implicated in the pathophysiology of fibromyalgia, with a decrease in dopamine concentration leading to central pain sensitization [83]. There may also be a correlation between the decrease in dopamine levels and other symptoms of fibromyalgia such as fatigue, sleep disturbances and depression. This has raised interest in direct or indirect dopaminergic actions as a potential mechanism for the treatment of fibromyalgia. Although amitriptyline has been suggested to exhibit limited effects on the dopaminergic system, the combination of amitriptyline and fluoxetine as a treatment of fibromyalgia was suggested to offer a preferable serotonin/noradrenaline/dopamine uptake inhibition than what was provided by the individual drugs [32]. Sertraline and TD-9855 offer a reduction of the pain and fatigue levels, and they have a FIQ score similar to amitriptyline (Figure 1); however, they are more potent inhibitors of the dopamine reuptake transporter (Table 1). This additional property of sertraline and TD-9855 may enhance the symptom-relieving effects obtained with serotonergic and noradrenergic mechanisms of action. Bupropion has been reported to inhibit the dopamine reuptake transporters and NETs, but not the neuronal reuptake of serotonin [84]. A case study in a patient with fibromyalgia reported that bupropion improved pain scores and other fibromyalgia symptoms such as depression and cognitive function, over a two-month period [85]. Sibutramine, a serotonin/noradrenaline/dopamine reuptake inhibitor, has also been shown to reduce pain and fatigue, and increase overall functioning in patients with fibromyalgia [86]. Involvement of the dopamine system in the actions of amitriptyline as a treatment of fibromyalgia is worthy of further consideration.

A multimodal action of amitriptyline in fibromyalgia probably involves CNS and peripheral targets with contributory actions on receptors and ion channels in addition to monoamine reuptake inhibition.

## 4. Conclusions

Treatment of fibromyalgia remains a major unmet medical need and optimal translation of current research to the clinic has been limited. Nociceptive signalling involves a range of receptors, mediators, cells, and physiological and genetic changes supporting the need to target multiple events to evoke effective control of the symptoms of diverse patients with fibromyalgia. Consequently, drugs that act through diverse mechanisms and that are given as combination therapy are often standard for the treatment of fibromyalgia [4,5,6,87]. Several of the actions of amitriptyline have the potential to cumulatively interfere with, at least, the pain mechanism in fibromyalgia. Relative to other antidepressants (SNRIs, SSRIs, NRIs), TCAs could be more effective as analgesics because of their actions on multiple nociceptive and sensory targets at central and peripheral levels.

The heterogeneity of fibromyalgia has led to the proposal of the existence of subgroups of patients [88]; however, analgesic drug development is based on the mean responses of groups of patients with the same condition given the same drug and dose, with individual subjects or subgroups of the patient population given little consideration [89]. With such a heterogeneous condition, effectiveness is going to be dependent on multimodal pharmacology, as exhibited by amitriptyline. Understandably, involvement of a diverse pharmacology has the potential of increasing the incidence and profile of adverse effects which may lead many patients to discontinue use. Greater understanding of the role of the mechanisms of action of amitriptyline in the management of the symptoms of fibromyalgia requires further investigation to provide clues about the pathophysiology of the condition and to provide a preferable pharmacological profile for future drug development.

## Figures and Tables

**Table 1:** Summary of affinities of antidepressant drugs for monoamine reuptake transporters and neurotransmitter receptors. Data are presented as concentrations (nM) representing binding to the protein (Ki value) or exhibiting 50% inhibition of the protein function (IC_50_); where multiple values are available, a range of the published values is presented. For 5-hydroxytryptamine (5-HT) and histamine receptors, subtypes are indicated in parentheses after the data values. For receptor subtypes not stated, IC_50_ value was >10,000 nM. NET is the noradrenaline reuptake transporter; SERT is the serotonin reuptake transporter; DAT is the dopamine reuptake transporter. N/A = not available.

Drug	NET	SERT	DAT	Receptors				References
				Adrenergic	Muscarinic	Histamine	5-HT	
	Ki/IC_50_ nM	Ki/IC_50_ nM	Ki/IC_50_ nM	IC_50_ nM	IC_50_ nM	IC_50_ nM	IC_50_ nM	
Amitriptyline	13.3–63	3.13–67	2580–7500	24–690	7.2–26	1.1(H1), 1000(H3), 33.6(H4)	450(1A), 40(1B), 4(2A), 40(2B), 6(2C), 89–103(6), 126–398(7)	[10,11,12]
Citalopram	4870–>10,000	1.13–19.0	>10,000	560–1211	1430	180–286	617–6000(2)	[10,13,14]
Cyclobenzaprine	126	251	7943	10–110	8–30	1–6	20(2A), 200(2B), 62(2C), 40(6), 69(7)	[11]
Duloxetine	1.17–42	0.07–13	200–439	8300–8600	3000	2300	504(2A), 419(6)	[10,15,16,17,18,19]
Fluoxetine	563–1021	1.0–10	4180	3000	870–2700	3250	200(2A), 5000(2B), 73(2C)	[12,16,18,20]
Milnacipran	22–200	8.4–151	>100,000	>10,000	>10,000	>10,000	>10,000	[10,16,18,19]
Nortriptyline	1.49–8.3	16.5–317	1200–5000	55–2030	40–110	15.1(H1)	294(1A), 5(2A), 8.5(2C), 148(6)	[10,12]
Paroxetine	100–156	0.34–2	963	2741	72–340	>10,000	9034(2C)	[12,14,16,20]
Reboxetine	3–13.4	242–274	>10,000	>10000	6700	312	457(2C)	[12,14,16]
Sertraline	715–925	0.9–2.8	315	188	427	6578	2298(2C)	[18,20]
TD-9855	2–4	3–10	160–200	N/A	N/A	N/A	N/A	[21]
Trazodone	>10,000	367	>7000	153–728	>10,000	220(H1), 3290(H2)	118(1A), 106(1D), 36(2A), 78(2B), 224(2C), 1780(7)	[12]
Venlafaxine	538–2483	7.8–145	3070–7647	>10,000	>10,000	>10,000	2000(2), 2800(6)	[10,13,16,17,18,19]

**Table 2:** Efficacy of antidepressant drugs in the treatment of fibromyalgia. Data are presented based on the effects of antidepressant drugs on the Fibromyalgia Impact Questionnaire (FIQ), Patient’s Global Impression Assessment and primary symptoms. Changes in score ratings are presented on a 0 to 10 scale, except FIQ (0–100), Hamilton Rating Scale for Depression (HDRS), Hamilton Anxiety Rating Scale (HAM-A; 0–56), Beck Depression Inventory (BDI; 0–63), Beck Anxiety Inventory (BAI; 0–63), and Montgomery–Åsberg Depression Rating Scale (MADRS; 0–60). The proportion (%) of patient population gaining significant benefit for a domain, where available, is indicated by ^#^. MOS = Sleep Scale from the Medical Outcomes Study; VLD = very low dose.

Drug	Dose (mg/day)	FIQ	Patient’s Global Assessment	Symptoms				References
				Pain	Fatigue	Sleep	Anxiety and Depression	
Amitriptyline	10–100	−13 to −19	−0.5 to −3.8 87% ^#^	−0.4 to −5.3	−2.2 to 3.5 80−93% ^#^	−1.1 to −3.9 63−100% ^#^	HDRS −9.9 to −10.4 BDI −6.2 to −7.0 50% ^#^	[29,30,31,32,33,34,35,36,37,38,39,40,41]
Citalopram	20–40		52.9% ^#^	−1.2		−0.59	MADRS −4.0	[42]
Cyclobenzaprine (TNX102SL)	10 VLD 1–4			−0.9	No effect	Parameters improved 12.3−38.5%	Improved 24.1%	[43,44,45,46,47]
Duloxetine	30–120	−7.96 to −18.4	−2.79 to −3.43	−1.6 to −2.4	−0.33 to −3.8	−2.67 to −2.69 Caused insomnia	BDI −3.32 to −5.47 HDRS −2.04 to −7.8	[48,49,50,51,52,53,54,55,56]
Esreboxetine	4–10	−3.9 to −7.2	40−42.6% ^#^	−0.4 to −0.76	−0.59 to −0.64	Caused insomnia		[57]
Fluoxetine	10–80	−10.9 to −11.5	−1.77	−2.3 to −2.4	−1.6	−0.86	No effect	[32,58]
Milnacipran	30–200	−12.3 to −26	35−51% ^#^	−1.6 to −3.5	−4.3 to −7.3	No effect	BDI −2.1 to −4.9	[59,60,61,62,63]
Paroxetine	12.5–62.5	−6.6 to −6.8	45−55% ^#^	−0.95		−4.6 to −4.9	BDI −4.6 to −5.8	[64,65]
Sertraline	50		83% ^#^	−4.2		MOS Score −15		[66]
TD-9855	20	−16.2	48% ^#^	−1.4		Caused insomnia		[67]
Trazodone	50–300	−9.6 to −13.4		−0.52 to −1.41	−0.72 to −1.17	−4.2 to −5.0	HDRS −1.4 to −2.0 HAM-A −1.5 to −2.4 BDI −5.5 to −8.9	[68]
Venlafaxine	75–300	−9.0 to −19.9	51% ^#^	−1.87 to −2.14	35% ^#^		HDRS −3.6 to −4.65 HAM-A −7.14 to −13.5 BAI −8.5 BDI −7.9	[34,69]
